# Quantifying the resilience of rapid transit systems: A composite index using a demand-weighted complex network model

**DOI:** 10.1371/journal.pone.0267222

**Published:** 2022-04-28

**Authors:** Hong En Tan, Jeremy Hong Wen Oon, Nasri bin Othman, Erika Fille Legara, Christopher Monterola, Muhamad Azfar Ramli

**Affiliations:** 1 Systems Science Dept, Institute of High Performance Computing, A*STAR, Singapore, Singapore; 2 Analytics, Computing, and Complex Systems Laboratory, Aboitiz School of Innovation, Technology, and Entrepreneurship, Asian Institute of Management, Makati, Phillippines; University of Ottawa Faculty of Engineering, CANADA

## Abstract

Quantifying the impact of disruptions on rapid transit resilience is crucial in transport planning. We propose a composite resilience score for rapid transit systems comprising four indicators that measure different physical aspects of resilience. These are computed using a weighted network model incorporating the network structure of stations, differences in line capacities, and travel demand. Our method provides a holistic assessment of network resilience and allows for straightforward comparisons of different scenarios including rail expansions and changes in demand. Applying our methodology to multiple configurations of Singapore’s rapid transit system, we demonstrate its effectiveness in capturing the impact of planned future lines. We also showcase through simulated studies how tipping points in resilience arise when demand varies. Furthermore, we demonstrate that system resilience could be unintentionally reduced by redistributing commuting demand to peripheral areas. Our methodology is easily applied to other rapid transit systems around the world.

## Introduction

Transportation systems are the lifeblood of cities; worldwide, they are acknowledged as crucial factors for healthy economies and stable societies [1]. Rapid transit systems (RTS), also known as metro or subway systems, are especially crucial due to their ability to sustain higher passenger throughput as compared to other public transport modes such as buses. RTS also has the advantage of operating independently from the road network infrastructure thus reducing traffic congestion and lowering the city’s carbon footprint. Having an efficient RTS enhances both mobility and accessibility within urbanized systems, and indirectly furthers economic advancement [2]. With rapid urbanisation and continued growth of cities, RTS therefore plays an increasingly significant role in serving the growing transportation needs of the public. For example, Singapore adopts a hub-and-spoke public transport model where the RTS acts as the backbone of the system, and buses fulfill the first and last mile transportation. As of 2016, its RTS catered to over three million trips per day, and ridership is expected to grow further as coverage expands.

However, a heavy reliance on the RTS as the primary transport mode in the city means that service disruptions could severely impact the entire city if not managed correctly. The effects are especially pronounced during a prolonged disruption, where failure in an initially localised region could lead to service degradation upstream, to other lines in the RTS system, or worse, other modes of transportation. Without proper contingency plans, service disruptions could cause these cascades of travel disruption throughout the transportation system [3] that ultimately engenders significant economic costs to the city.

As such, transport planners and operators not only have to strive to minimise the likelihood of disruptions, they also need to consider the ability of the RTS to withstand these events. In anticipation of potential service disruptions, planners tend to design with additional redundancies built in the system in order to ensure the availability of alternative routes for regular commuters. However, significant costs are incurred in building and operating additional RTS infrastructure [4] so a comprehensive cost benefit analysis that incorporates resilience is required. This quantitative understanding of the resilience of the system can help transport planners better understand vulnerable edges in the RTS, and provide a new dimension that goes beyond sole economic benefit, assisting them in making decisions when upgrading or improving RTS networks.

In contrast to previous works that typically approach metro resilience from one aspect only such as network topology or passenger flow, the first and primary objective of this paper is to propose a composite resilience score for RTS networks comprising four indicators measuring different physical aspects of resilience. These indicators measure the importance of an edge, defined as the link or rail connection between two metro stations, with regards to maintaining functionality of the network should that edge become disrupted. In this work, the indicators measure the impact of disruptions on the shortest paths, accessibility of alternative stations, amount of additional travel time incurred and availability of spare capacity on trains to accommodate redirected commuters. By considering the different aspects of resilience, our method provides a holistic assessment of RTS resilience and also allows for comparisons of different scenarios including rail expansions and changes in demand. The second objective of this paper is to apply our methodology on a real network, using Singapore’s RTS as a case study. We applied our methodology to past and planned future network configurations and demonstrate its effectiveness in capturing the changes in overall system resilience brought about by increased commuter population and opening of new lines. However, a main limitation that we would like to highlight is that our current framework does not yet take into consideration the role of other public transport services such as public buses or taxis in helping to alleviate RTS service disruptions. This is because in its current state, the measure we proposed is primarily meant to assess and quantify the resilience characteristics of the rapid transit system on its own rather than an attempt to quantify the resilience of the entire public transportation network. However, due to the modular design, resilience considerations involving alternative modes of transports could be potentially incorporated into this framework in the future. This can be done by designing additional indicators that utilize information from other transport networks that could be included in the overall aggregated resilience index.

The rest of the paper is organised as follows: We first review the definitions of resilience and concepts related to resilience, as well as work that has been done to quantify and measure resilience in transportation systems. Subsequently, we introduce our concept of resilience as explored within the context of this paper. We then define the network model that will be used throughout the remainder of the article and mathematically define a composite index score that comprises four indicators and identify the different resilience aspects that they represent. Subsequently, we demonstrate the application of our methodology on the RTS of Singapore including scenario studies that demonstrate how commuting demand affects the resilience of the system. Finally we conclude the article by describing the limitations of our method, providing a discussion on possible extensions of the model and finally summarising the main findings of the paper.

## Literature review

In previous works found in recent literature, terms such as ‘robustness’ and ‘resourcefulness’ have been used to describe the qualities of a resilient system, while terms such as ‘vulnerability’ and ‘susceptibility’ has been used to describe the lack of resilience in a system. Here we describe the relevant literature which help define resilience and its relation to these qualities in the context of our article.

### Definitions and aspects of resilience

The available literature defines resilience in two distinct ways. The first defines resilience of a system as a measure of the speed with which the system returns to equilibrium after being subjected to a perturbation [5], while the second interprets resilience in terms of a system’s capability to persist when exposed to changes or shocks [6]. Rose [7] summarised the above definitions respectively: a) dynamic or engineering resilience refers to “speed at which an entity or system recovers from a severe shock to achieve a desired state”, while (b) static or ecological resilience is defined by the “ability of an entity or system to maintain function when shocked”. To put it simply, static and dynamic resilience can be thought of as qualities of a system pertaining to stages during and after disruptions respectively.

In work related to the assessment and enhancement of resilience during seismic events [8], the authors further expanded upon these resilience qualities and categorised them into four dimensions: robustness, redundancy, resourcefulness, and rapidity. They are defined as follows:

1. Robustness: strength of a system to withstand a given level of stress without suffering degradation or loss of function.
2. Redundancy: the extent to which an element in the system can be substituted such that the system is still capable of satisfying functional requirements in the event of a disruption.
3. Resourcefulness: the capacity to identify problems, establish priorities, and mobilize resources when disruptions occur; ability to apply material and resources to meet established priorities and achieve goals.
4. Rapidity: the capacity to meet priorities and achieve goals in a timely manner in order to contain losses and avoid future disruption.

One aspect of resilience not considered in [8], but is relevant to transportation networks, is that of reliability. Reliability is commonly used to describe the stability, certainty, and predictability of travel conditions and can be defined in terms of connectivity (probability of reaching destination), travel time (probability of reaching the destination by a given time) [9], and capacity reliability (probability of network to accommodate a certain amount of traffic) [10].

From these definitions of robustness, reliability, and redundancy, we see clear similarities to the definition of static resilience as they involve measuring the ability of a system to persist or maintain function during a disruption. On the other hand, rapidity has a more obvious relationship to dynamic resilience as it involves a time-to-recovery and post-disruption aspect. Resourcefulness has elements of static and dynamic resilience as the qualities it represents can aid the system both during and after the disruption.

Finally, the vulnerability of a transport system can be understood as the susceptibility of the system to incidents that can result in considerable reductions in functionality [10], and can be treated as the converse of resilience to a certain extent [11]. Resilience pertains more to conditions and responses that happen during or after a disruption, while vulnerability concerns mainly pre-disruption conditions [7, 12]. Different authors focused on different aspects of resilience and in the subsequent section, we examine how these resilience aspects are quantified and measured in previous studies pertaining to rail transportation.

#### Related work

Metro networks could be described in terms of general topological and geometric features in contrast to localised, city-specific features. In [13], the authors proposed measures for characterising network size and form, as well as indicators for assessing network topology such as connectivity and directness, under the mathematical framework of graph theory. This was expanded upon in [14], who applied previously developed network indicators [13, 15, 16] to evaluate and assess the performance of 13 metro networks in terms of topology, width and density, and territorial coverage. These network indicators that were formerly used for characterising performance and structure of the network [17] became more commonly used to study and quantify the robustness of these networks [18].

In [19], the authors proposed three resilience measures based on throughput, connectivity and compactness, and applied these indicators to 17 theoretical graph networks that were extrapolated from real-life transportation networks. In general, they summarised that network resilience increased with graph features such as average node degree and cyclicity, but decreased with network diameter. Other work proposed ten theoretical and four numerical metrics to quantify the robustness of 33 metro networks by simulating targeted attacks and random failures [20]. The authors concluded that networks with large number of alternative paths and short overall path-lengths are generally more robust. Finally, researchers also assessed the vulnerability of metro networks in Shanghai, Beijing and Guangzhou in terms of the decrease in network efficiency (reciprocal of shortest paths) and measured the loss of functionality of the metro as the fraction of removed nodes is increased [21].

The primary drawback from the aforementioned literature is that they did not take into consideration the commuting load borne by different areas of the networks and as such, the vulnerability studies analysed and simulated were purely based on their network topology [22]. Past studies showed that network topology and the passenger flow is weakly correlated and highly sensitive to outliers [23], thus network topology alone might not provide a complete understanding of passenger flow patterns. On the other hand, a system-based approach incorporates data on passenger demand in order to capture the effect of differences in the distribution of commuter flow on network vulnerability.

Bell *et al* incorporated commuter flow into the analysis of network reliability [24] and vulnerability [25] using a novel game theoretic approach, where commuters and disruptions in the network are modelled as two non-cooperative players. The overall network reliability and vulnerability is then evaluated using the expected trip cost at the Nash equilibrium. Both in [26, 27], researchers used the Madrid metro as their case study, quantifying network robustness in terms of the total commuter travel time for all origin-destination (OD) pairs when edges in the network fail. The former group focused on how bus bridging services can ameliorate the effect of disruptions while the latter incorporated unsatisfied travel demand as another indicator for measuring vulnerability. Similar work was carried out by studying how bus services can be integrated with the Singapore metro network to reduce unsatisfied passenger demand in the event of edge disruption [28]. In [29], they took a slightly different approach towards quantifying robustness and redundancy in Stockholm’s metro by calculating changes in overall passenger welfare—defined in terms of a generalised cost function—when the network structure is altered either due to disruptions or by rail expansion projects. In [30], Tabu Search was utilized to optimize different rail line alignments for the Shanghai Metro using a similarly computed cost function.

This framework was also used in work where authors investigated the influence of line capacity on passenger welfare for the metros of Stockholm and Amsterdam respectively [31–33]. Another group approached metro resilience differently by defining resilience in terms of the speed at which a metro network can recover after experiencing a disruption [34]. They measured recovery rate using passenger count data and applied their findings on the London Underground. In [35], robustness of the London metro was also quantified according to how overall passenger flow and node-pair connectivity are impacted as the number of nodes are removed while in [36], they measured vulnerability by removing entire lines instead of single stations so as to reflect more accurately the nature of disruptions in his case study of the Shanghai metro network. Finally, in [37], they used a dynamical system approach to model cascading failures in the Beijing rapid transit network using a coupled map lattice approach. They also analyzed the effect of different thresholds and concluded that circular or loop-based lines were generally more vulnerable as compared to linear radiating rail lines.

In summary, resilience is a multifaceted concept which results in many different interpretations and approaches to quantify it. Most of the previous studies focused mainly on a single particular aspect of resilience that is usually derived from the topological structure of the metro network. This may not provide a complete picture of overall system resilience. We bridge this gap by considering other aspects of the metro network, in particular, availability of alternative routes and spare capacity, proximity to other stations, effect of commuter flow, and incorporating these factors into our resilience formulation. In the next section, we lay out the scope and framework for defining and quantifying different aspects of resilience in a rail network.

## Methodology

### Resilience framework

Our approach to analysing rail resilience leans more towards static or ecological resilience, where we attempt to quantify and measure the ability of the system to persist or maintain functionality after being perturbed [6, 7]. This is because static resilience is important for long term planning of network design and operations. Furthermore, information such as travel time and train occupancy that can be inferred from ticketing data allows us to compare and measure the effect of disruptions on the ability of the system to maintain functionality, through the computation of metrics such as additional travel time, additional distance travelled, number of affected commuters, and the availability of feasible alternative routes using a weighted network model. Since information derived from ticketing data such as commuter flows and train frequencies will change depending on the time of day, some of these computed metrics will also exhibit a temporal dependence.

Two types of disruptions are employed within our resilience methodology:

1. A complete disruption means that no commuter is allowed to traverse through the specified edge and will therefore need to find an alternative route (sometimes there are no alternative routes) to get to his/her specified destination.
2. A partial disruption is modelled by imposing a cost penalty (additional travel time) when commuters are required to traverse the edge.

The scenarios that are defined in our indicator definitions could also involve either a single edge, or a set of edges (typically grouped within a single line) being disrupted. We also currently assume that during the disruption, all commuters will continue to attempt to complete their journeys on the RTS network without resorting to other modes of transport given constraints on available train capacity. We assume that commuters are also not provided adequate advanced warning to change their proposed travel. Similarly as above, our scenario assumes no additional mitigation measures (such as bus bridging services) are provided by transport operators during the disruption period. While we recognize that the scenario is not fully reflective of the situation in an actual train disruption, our approach can be justified by understanding that our particular resilience measure reflects the worst-case scenario where commuters are forced to rely solely on the RTS network.

### Weighted flow network of a rapid transit system

We represent the RTS as a directed graph denoted by *G* = (*V*, *E*), where *V* and *E* are the sets of vertices and edges respectively. The vertices *v* ∈ *V* represent the train stations while the edges *e* = (*v*_*i*_, *v*_*j*_)∈*E* represent the rail connection between two consecutive stations. Let the matrix *M* = (*m*_*ij*_) represent the commuting demand for all origin-destination (OD) pairs, where the matrix element *m*_*ij*_ denotes the number of passengers going from *v*_*i*_ to *v*_*j*_. The demand matrix can either be obtained from either empirical data [38] or from various demand forecasting methods [39–41]. Let *N* denote the total number of stations in the network, the total number of OD pairs is then given by *Z* = *N*(*N* − 1). This quantity will be used as a normalising factor for one of the indicators in the subsequent section.

In general, there may be more than one alternative path joining two nodes together. We denote *r*_*k*_(*O*, *D*) as the *k*^th^ alternative route from an origin node *O* to a destination node *D*, and it is defined as the ordered set of edges that join these two nodes together:

$$\begin{array}{c} r_{k}\left(O,D\right)=\left\{\left(O,v_{1}\right),\left(v_{1},v_{2}\right),...,\left(v_{i},D\right)\right\}. \end{array}$$ (1)

Let *τ*(*e*) be the time taken to traverse edge *e*. The time taken to travel from from *O* to *D* via route *r*_*k*_ is given by

$$\begin{array}{c} \tau (O,D;r_{k})=\tau \left(O,v_{1}\right)+\tau \left(v_{1},v_{2}\right)+...+\tau \left(v_{i},D\right) \end{array}$$ (2)

$$\begin{array}{c} =\sum_{e^{\prime }\in r_{k}}\tau \left(e^{\prime }\right). \end{array}$$ (3)

In previous studies, it is assumed that commuters take the path with the shortest travel time [26, 42, 43]. However, this simple assumption has been shown to be inadequate when estimating the network flow distribution. In order to assign the appropriate number of commuters more accurately, we modelled commuters’ choice behaviour using random utility theory [44]. In this framework, the true utility *T*(*O*, *D*;*r*_*k*_) of a particular route *r*_*k*_ is expressed as a sum of an observed utility *U*(*O*, *D*;*r*_*k*_), and a randomly varying error term *ϵ*(*O*, *D*;*r*_*k*_) for the unobserved utility. In our formulation, the observed utility associated with choosing route *r*_*k*_ is modelled as a linear function of travel time (*T*), number of transfers (*R*) and number of stations (*S*) along route *r*_*k*_:

$$\begin{array}{c} U\left(O,D;r_{k}\right)=\beta_{T}T\left(r_{k}\right)+\beta_{R}R\left(r_{k}\right)+\beta_{S}S\left(r_{k}\right). \end{array}$$ (4)

We performed a multinomial logistic regression using maximum likelihood estimation to fit the *β*-coefficients of the utility function to the different routes [45] observed in empirical data. If the error terms are assumed to be independently and identically distributed according to a Weibull distribution, then the choice probability for route *r*_*k*_ can be written as [46]:

$$\begin{array}{c} P\left(r_{k}|O,D\right)=\frac{\text{exp}(U\left(O,D;r_{k}\right))}{\sum_{r_{l}\in R^{OD}}\text{exp}\left(U\left(O,D;r_{l}\right)\right)}, \end{array}$$ (5)

where *R*^*OD*^ = {*r*_1_, *r*_2_, …, *r*_*K*_} is the set of all alternative routes for that OD pair.

The flow of passengers along a particular route *r*_*k*_ for an OD pair can therefore be expressed as:

$$\begin{array}{c} f\left(O,D;r_{k}\right)=P\left(r_{k}\;|\;O,D\right)\times m_{OD}. \end{array}$$ (6)

The overall commuter flow across a single edge *e* in the network is therefore the sum of flows for all routes that pass through the edge over all the OD pairs as described by the following equation:

$$\begin{array}{cc} & f\left(e\right)=\sum_{O,D\in V,O\neq D}\;\sum_{r_{l}\in R^{OD}}\;\chi_{r_{l}}\left(e\right)\;f\left(O,D;r_{l}\right), \end{array}$$ (7)

$$\begin{array}{cc} & \chi_{r_{l}}\left(e\right)≔\{\begin{array}{c} 1\;\text{if}\;\;e\in r_{l},\\ 0\;\text{if}\;\;e\notin r_{l}. \end{array} \end{array}$$ (8)

where $\chi_{r_{l}}\left(e\right)$ is an indicator function for the membership of edge *e* in route *r*_*l*_.

It should also be noted that our definition of edge passenger flow differs from typical road traffic flow (which refers to vehicles per unit time) used traditionally by the transportation field.

With these basic definitions in place, we go on to define four proposed indicators that make up the resilience index. Since the RTS network is represented by a directed graph, computation of the indicators would be carried out for all edges separately to account for asymmetric flows that may occur between two stations. To ensure that these indicators could eventually be aggregated or decomposed easily, we intentionally designed them such they all shared the following properties:

1. Each indicator is a function of the attributes of an edge.
2. Every individual indicator score is normalized between 0 (resilient) and 1 (not resilient).

This ensured the following valuable features: a) Easy comparison of scores between different edges within the same scenario configuration to highlight areas of low resilience in the network. b) Combining indicator values are easily done by taking a weighted average based on relative importance. 3) Edge comparisons can easily be conducted even between different network configurations and scenarios. More examples of this are provided in the Results section. The remainder of this section describes each indicator in detail and the weighting scheme used to compute the aggregate index.

### Indicator definitions

#### Betweenness centrality

The first indicator we introduce is a commonly used measure in network science and graph theory, known as betweenness centrality (BC) [47]. In particular, we utilize the edge betweenness centrality to ensure consistency with the required property (1) listed in the earlier section. It is straightforward to understand why this indicator is commonly associated with vulnerability and resilience, as edges with high BC values imply that a large number of shortest paths between different network nodes utilise that particular edge, making it a critical component if it was to be disrupted. Mathematically, the normalised form for directed graphs is written as:

$$\begin{array}{c} BC\left(e\right)=\frac{1}{N(N-1)}\sum_{O,D\in V,O\neq D}\frac{\sigma (O,D\;|\;e)}{\sigma (O,D)}, \end{array}$$ (9)

where *N* is the total number of stations, *σ*(*O*, *D*) is the total number of shortest paths connecting origin station *O* to destination station *D*, while *σ*(*O*, *D*|*e*) is the number of those paths that pass through edge *e*.

It should be noted that BC is a graph theoretic measure that is based purely on network topology. On one hand, it can be easily used to compare between networks of various cities [48] without requiring network specific information such as passenger, train scheduling, train capacity data. However, certain nuances such as the inequivalence of commuter demand at stations is overlooked when computing this topological quantity. This is especially true in the context of Singapore’s RTS, where there are two distinct types of services in the network—the Light Rapid Transit (LRT) or light rail service and the main Mass Rapid Transit (MRT) service. The LRT trains are essentially smaller versions of the MRT trains, both in terms of carrying capacity and train speeds. Their role is to act as a feeder service for the MRT lines, shuttling commuters from housing estates to the main MRT stations. By using Eq 9 to compute the BC of the full network, segments of the network that join up to the LRT lines are unfairly assessed due to the increased number of OD pairs associated with the LRT stations even though the demand for each individual LRT station is much lower compared to a MRT station.

Rather than removing LRT stations which causes fragmentation of the network, we address this issue by weighting each OD pair in the BC computation according to the maximum carrying capacities of trains incident at the origin and destination stations. The capacity-modified form of edge BC becomes

$$\begin{array}{c} BC_{cap}\left(e\right)=\frac{\sum_{O,D\in V}\frac{\sigma (O,D\;|\;e)}{\sigma (O,D)}C\left(O\right)C\left(D\right)}{\sum_{O,D\in V}C\left(O\right)C\left(D\right)}, \end{array}$$ (10)

where *C*(*i*) is the train carrying capacity at station *i* computed by averaging the train capacities along the edges connected to station *i*. With such a modification, shortest paths between LRT stations will be given less weightage compared to paths between MRT stations but the downside is that additional network information regarding train capacity is required.

Even though the normalised form of BC given in Eq 9 is bounded in the interval (0, 1) and there is a physical meaning associated with it (proportion of shortest paths), it is less clear when it comes to assigning a resilience score to the BC values as there is no basis for comparison. Here, we propose a second modification to the BC indicator by comparing it against the BC of a worst case theoretical network—the linear chain with the same number of nodes. For any (directed) tree graph with *N* vertices and 2(*N* − 1) edges, it can be shown (see S1 Appendix in S1 File) that the linear chain has the largest average network BC given by

$$\begin{array}{c} {\bar{BC}}_{0}=\frac{N+1}{6(N-1)}. \end{array}$$ (11)

This implies that the linear chain is the least resilient in terms of random disruptions along the edges as it will result in the highest proportion of unsatisfied OD journeys. The computed BC values are scaled by this value according to $BC^{\prime }\left(e\right)=BC_{cap}\left(e\right)/{\bar{BC}}_{0}$. A value of *BC*′ that is close to 1 implies that the metro network is very similar to a linear chain network in terms of the distribution of shortest paths, which negatively impacts the resilience of the metro network in terms of unsatisfied OD trips.

#### Nearest transport point

Metro networks in larger more developed cities generally consist of more than a single lines, usually distinguished by name, colour, number, or a combination of the above. Individual train lines are also typically serviced by a different fleet of trains and commuter wanting to travel on multiple lines usually requires some form of transfer from one train to another. As train disruptions occurring along a specified edge usually also only affects the particular line associated with that edge, our formulation of the nearest transport point (NTP) indicator evaluates the geographical distance from a particular edge to the nearest edge belonging to another line simulating the scenario where a commuter is able to take an alternative line in order to complete their journey. An edge that is in close proximity to other lines is therefore arguably more resilient to disruptions as commuters have viable alternative routes through either taking a short bus or bike trip, or even walk in order to relocate to another station compared to an edge that is far away from other lines.

The calculation of NTP involves iterating through all the edges *e* = (*v*_*i*_, *v*_*j*_) in the network, evaluating the geographical distance from the origin node *v*_*i*_ of edge *e* to the origin node $v_{i}^{\prime }$ of another edge $e^{\prime }=\left(v_{i}^{\prime },v_{j}^{\prime }\right)$ that belongs to the nearest other line. If this distance *d*(*e*, *e*′) is more than a certain threshold distance *δ* away, then that edge is penalised. Mathematically, the NTP indicator for edge *e* is expressed as:

$$\begin{array}{c} NTP\left(e\right)=\{\begin{array}{cc} 0\;, & d(e,e^{\prime })<\delta \\ \frac{d(e,e^{\prime })-\delta }{\delta }\;, & \delta \leq d(e,e^{\prime })\leq 2\delta \\ 1\;, & d(e,e^{\prime })>2\delta . \end{array} \end{array}$$ (12)

By writing NTP in this form, edges that are very accessible (less than one unit of threshold distance away) are given a score of 0; edges that are not accessible (more than 2 units of threshold distance) are given a score of 1; while edges that lie between the ends of the spectrum are given a score between 0 and 1 that scales linearly with *d*.

#### Passenger delay

In the event of a disruption across an edge, passenger flow across that edge would have to be redirected via alternative routes within the RTS system, if they exist, in order for the commuters to reach their destinations. The quality of the alternative routes is quantified using two indicators: passenger delay (measuring the extra travel duration) and vulnerable passenger flow (measuring the amount of spare train capacity available on these alternative routes). Since these indicators depend on train frequency, their values would change depending on the time of day. For example, the average train frequency during the morning peak period is usually higher than the off-peak period in the early afternoon. The passenger delay indicator will be introduced in this section and vulnerable passenger flow indicator will be described in the next section.

The passenger delay (PD) indicator for an edge measures the average extra travel time experienced by commuters when that edge is disrupted. This indicator not only evaluates the existence of alternative routes, but also quantifies the feasibility of these routes in terms of the extra journey time required. Thus, a resilient network is one that not simply has alternative routes for commuters to get to their destinations when a disruption occurs, but that these routes do not add unreasonably long travel times to the journeys.

We simulate an edge disruption by adding a time penalty *τ*_*p*_ to the total travel time when traversing through that edge, and the alternative paths for affected commuters are recomputed using a top-*k* shortest path algorithm. If no alternative routes exist, or if there are no better options than staying to the original route and waiting out the disruption, then that particular edge is not robust to disruptions.

Let *A*_*e*_ and *U*_*e*_ be the set of OD pairs that are affected and unaffected by the disruption of edge *e* respectively. An OD pair (*O*, *D*) belongs to set *A*_*e*_ if any of its alternative routes traverses the disrupted edge *e*; otherwise, it belongs to set *U*_*e*_. Mathematically,

$$\begin{array}{cc} \left(O,D\right)\in A_{e} & \text{if}\;\;\exists \;e\in r_{l}\left(O,D\right)\;\forall \;l,\\ \left(O,D\right)\in U_{e} & \text{otherwise}. \end{array}$$ (13)

The PD indicator for edge *e* is expressed as:

$$\begin{array}{c} PD(e)=\frac{1}{Z}\sum_{\text{all}(O,D)}\frac{\tau^{\prime }\left(O,D\right)-\tau_{0}\left(O,D\right)}{\tau_{p}} \end{array}$$ (14)

where *Z* is the total number of OD pairs in the undisrupted network and *τ*_0_(*O*, *D*) is the original duration of travel from *O* to *D* without disruption, and *τ*′(*O*, *D*) is the average duration of travel via alternative routes when *e* is disrupted, written as:

$$\begin{array}{c} \tau^{\prime }\left(O,D\right)=\sum_{r_{l}\in R^{OD}}\tau \left(O,D;r_{l}\right)\times P\left(r_{l}\;|\;O,D\right)\;, \end{array}$$ (15)

where *τ*(*O*, *D*;*r*_*l*_) and *P*(*r*_*l*_|*O*, *D*) are defined in Eqs 3 and 5 respectively. Here, we have assumed that the effects of the disruption are localised to edge *e* and that the alternative routes are functioning just as well as in normal conditions.

For OD pairs that are unaffected by the disruption at edge *e*, *τ*′ would be equal to *τ*_0_, and the contribution to the PD indicator for these OD pairs would be zero. Thus, these OD pairs can be omitted from the summation and the PD indicator can be rewritten as:

$$\begin{array}{c} PD(e)=\frac{1}{Z_{e}}\sum_{\left(O,D\right)\in A_{e}}\frac{\tau^{\prime }\left(O,D\right)-\tau_{0}\left(O,D\right)}{\tau_{p}}, \end{array}$$ (16)

where *Z*_*e*_ is the total number of OD pairs affected by disruption of *e* (i.e. number of OD pairs in set *A*_*e*_).

As the largest value of *τ*′ is given by *τ*_0_ + *τ*_*p*_ when waiting out a disruption, the PD value is guaranteed to be bounded in the interval (0, 1). An indicator value close to 0 implies that the network is resilient in terms of providing commuters with alternative paths that do not incur much additional travel time.

#### Vulnerable passenger flow

When passengers affected by a disrupted edge are redirected via alternative routes, the influx of these passengers puts a strain on the train capacity of the other lines. The vulnerable passenger flow (VPF) indicator quantifies how robust a metro network is in coping with such spill-over congestion when an edge is disrupted. The VPF of an edge *e* is defined as the fraction of affected passengers who cannot be accommodated on alternative travel routes due to limited train capacities when *e* is disrupted.

When an edge *e* is disrupted, passenger flow will need to be redirected into various other neighbouring edges *e*′ in order for the network to continue supporting the total flow demand. The total flow of passengers *f*(*e*′) across all other edges *e*′ in the network now comprises passengers unaffected by the disruption *f*_*u*_(*e*′), and passengers affected by the disruption *f*_*a*_(*e*′), given by:

$$\begin{array}{cc} f(e^{\prime }) & =f_{u}\left(e^{\prime }\right)+f_{a}\left(e^{\prime }\right),\\ f_{u}\left(e^{\prime }\right) & =\sum_{\left(O,D\right)\in U_{e}}\;\sum_{r_{l}\in R^{OD}}\;\chi_{r_{l}}\left(e^{\prime }\right)f\left(O,D;r_{l}\right), \end{array}$$ (17)

$$\begin{array}{c} f_{a}\left(e^{\prime }\right)=\sum_{\left(O,D\right)\in A_{e}}\;\sum_{r_{l}\in R^{OD}}\;\chi_{r_{l}}\left(e^{\prime }\right)f\left(O,D;r_{l}\right). \end{array}$$ (18)

The spare capacity *C*_*s*_(*e*′) of an edge is obtained by taking the difference between the total carrying capacity *C*_*t*_(*e*′) and unaffected passenger flow *f*_*u*_(*e*′):

$$\begin{array}{c} C_{s}\left(e^{\prime }\right)=\{\begin{array}{cc} C_{t}\left(e^{\prime }\right)-f_{u}\left(e^{\prime }\right)\;, & C_{t}\left(e^{\prime }\right)>f_{u}\left(e^{\prime }\right)\\ 0\;, & \text{otherwise}, \end{array} \end{array}$$ (19)

where *C*_*t*_(*e*′) is computed by multiplying the average hourly train frequency by the carrying capacity of a train.

The total number of vulnerable passengers is defined as the largest number of passengers that exceed the spare capacity along a path, summed over all alternative paths over all affected OD pairs. Mathematically, this is written as:

$$\begin{array}{c} \rho_{\text{vul}}\left(e\right)=\sum_{\left(O,D\right)\in A_{e}}\;\sum_{r_{l}\in R^{OD}}\;\text{max}\left\{f\left(O,D;r_{l}\right)-C_{s}^{\text{min}}\left(r_{l}\right),\;0\right\}, \end{array}$$ (20)

where $C_{s}^{\text{min}}\left(r_{l}\right)$ is the lowest spare capacity among the edges of route *r*_*l*_. Eq 20 basically counts the largest overflow of commuters due to a disruption in *e*.

Finally, since all commuters flowing across *e* would be affected by the disruption of *e*, the total number of affected commuters is simply *f*(*e*), and the VPF indicator is written as:

$$\begin{array}{c} VPF\left(e\right)=\frac{\rho_{\text{vul}}\left(e\right)}{f(e)}. \end{array}$$ (21)

An edge with a VPF value that is close to 0 means that commuters going through that edge have alternative routes with sufficient capacity to accommodate them should a disruption occur along that edge. Conversely, a value close to 1 means that either there are no possible alternative routes or the routes are already fully utilised with little spare capacity available.

### Aggregation of indicators

From research literature, commuter flows within the network is an important factor that should be considered for a more complete description of metro resilience [22, p.31]. In our formulation thus far, each indicator was designed with an independent property in mind, BC, a network topology-based measure, NTP a distance-based measure, PD a time-based measure, while VPF is a train capacity based measure.

However, in attempting to combine and measure the resilience of the overall network using these individual indices computed on individual edges, we need to account for the differences in commuter flow distribution between these edges in order to provide a holistic picture of the overall resilience of the entire network. As such, in computing the overall network measure of each indicator, we use a weighted average using commuter flow across an edge as the weighting parameter. The justification being that indicator scores for edges with high passenger flow should be given greater consideration when comparing against indicator scores for edges with smaller flow. This weighted form of the indicator is expressed by the following formula:

$$\begin{array}{c} wI\left(e\right)=\frac{I(e)\times f(e)}{\sum_{e\in E}f\left(e\right)}, \end{array}$$ (22)

where *I* can be replaced by the indicator functions *BC*′, *NTP*, *PD*, *VPF* introduced in the previous sections and *f*(*e*) refers to passenger flow as first defined in Eq 8. Although this scheme slightly obfuscates the physical meaning conveyed by the indicator score, the flow-weighted indicator value allows us to evaluate the relative importance of edges based on the combined effect of commuter flow and indicator value of the edges. Furthermore, since commuter flow is time-dependent, this scheme would introduce a temporal aspect into the model as well.

The indicators can then be combined using a suitable aggregation function *F* to form the resilience index score *RI* for the entire metro network:

$$\begin{array}{c} RI=F(wBC^{\prime },wNTP,wPD,wVPF) \end{array}$$ (23)

where *wI* = ∑_*e*∈*E*_ *wI*(*e*) is the network-level score for indicator *I*.

For the case study of Singapore’s metro network, we use a simple average of the four indicators as the overall resilience score.

## Case study: Singapore rapid transit network

### Individual resilience indicator results

**Fig 1:**
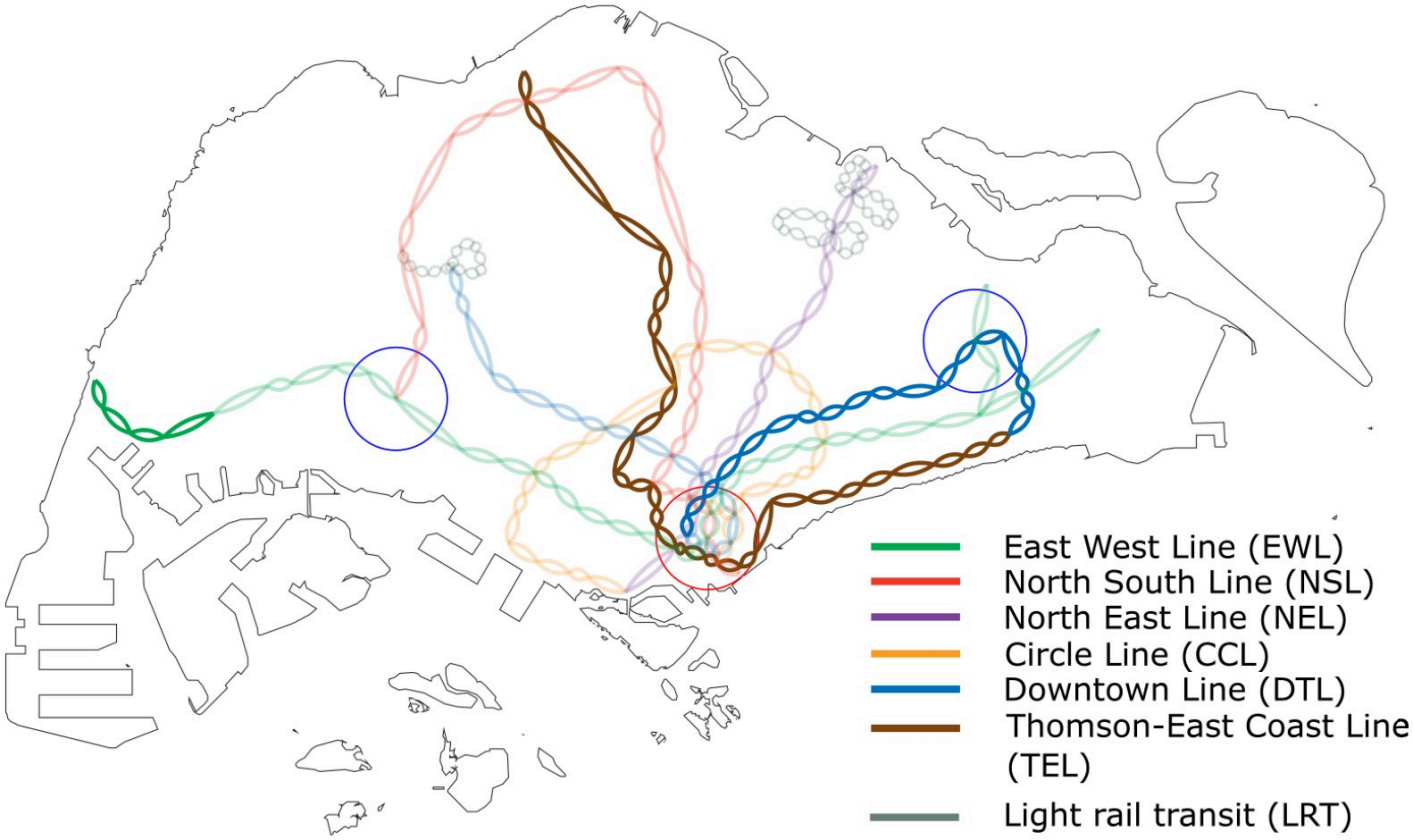
Map of Singapore’s rapid transit network. Links that already exist in the 2016 network are given a faint translucent colour while new links that will be built for the 2024 network are given a solid opaque colour. The red circle represents the central business district while the two blue circles represent regional centres that are planned for the eastern and western regions of Singapore. Names and abbreviations for the different rail lines are shown in the legend.

In this section, we demonstrate the application of our resilience framework on the RTS network of Singapore in 2016, and compare the results against a projected future network configuration in 2024. As of July 2018, the system currently operates five different MRT lines and three LRT lines (refer to Table 1 and Fig 1 for a reference to the different metro lines). By 2024, the full Downtown Line (DTL) as well as a new Thomson-East Coast Line (TEL) will be completed.

**Table 1:** RTS lines of the Singapore rapid transit network. Train frequencies for existing lines are based on present day scheduling information; TEL’s train frequency is assumed to be similar to that of the NEL.

RTS line name	Abbreviation	Frequency (hr^ − 1^)	Capacity *C*	Year of operations commencement
North South Line	NSL	17.1	1600	1987
East West Line	EWL	17.1	1600	1987
Light Rail Transit	LRT	17.1	150	1999 and 2005
Northeast Line	NEL	20.0	1600	2003
Circle Line	CCL	17.1	800	2009
Downtown Line	DTL	21.8	800	DTL1—2013 DTL2—2015 DTL3—2017 DTL3E—2024
Thomson-East Coast Line	TEL	20.0	1066	2024

By analysing past ticketing data, the demand matrix *M* for the 2016 network can be obtained. Due to the absence of such ticketing data for future networks, the 2024 demand matrix is estimated from land-use and population projections provided by our collaborators. For the computation of NTP, we used a threshold distance of *δ* = 2 km and the time penalty for computing PD is *τ*_*p*_ = 60 mins. These values were obtained through analysis of disruption events which happened in Singapore. For *δ*, this was obtained by observing regular commuters who changed their origin or destination stations as a result of the disruption and measuring the average distance between their original undisrupted station to the newly chosen station. For *τ*, similarly, we observed that one hour served as a good estimate for the average period of disruption typical for Singapore during the period of study.

For the remainder of the paper, we will be presenting results from the morning peak period taken from 7 to 9am, however the framework can be applied to other time intervals as well. The morning peak is chosen for this analysis as we believe that the metro system would be placed under greater stress by commuters rushing to get to work on time and this worst-case scenario would provide a better representation of the overall network resilience.

In each of the figures, circles represent the locations of the metro stations. The bidirectional edges connecting the stations are represented by loops pointing in a clockwise direction. The colour of an edge indicates the value of the indicator for that particular edge, while the thickness represents the flow of passengers across that edge. The flow-weighted average for the indicator computed over the entire network is shown in the legend.

**Fig 2:**
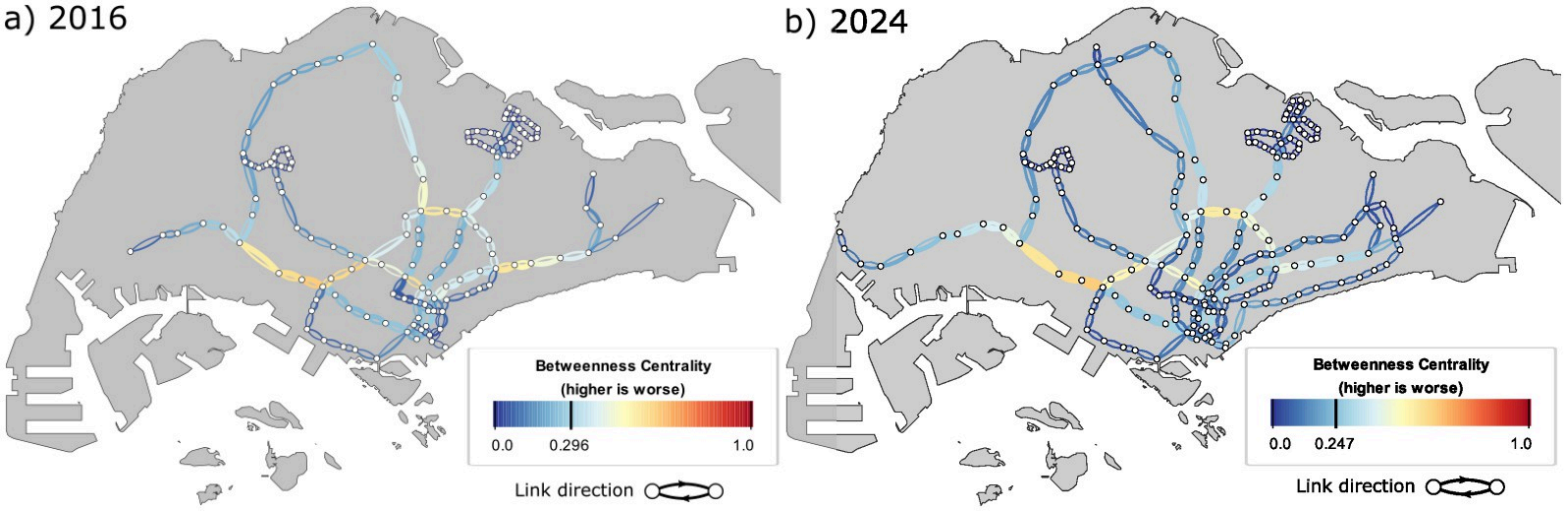
Results of the modified betweenness centrality (BC) indicator. a) 2016 and b) 2024, Edges with high BC are located between intersections of radial lines (EWL, NSL, and NEL) with the circle line (CCL). The overall flow-weighted network score is shown in the legend.

The BC results are presented in Fig 2 for both networks in 2016 and 2024. We observe that the regions of high BC are intuitively located at the intersections between the main radial lines (EWL, NSL, and NEL) and the circle line (CCL). This is due to the fact that these edges provide essential bridging between the majority of OD pairs. The importance of these edges remain largely unchanged in spite of the addition of a new line (TEL) and the extension of an existing line (DTL) as many shortest routes between OD pairs still make use of the existing RTS lines. Nonetheless, the additional lines in the eastern part of Singapore provides alternative paths that redirect commuter flow, such that the overall flow-weighted BC for the network has been reduced.

**Fig 3:**
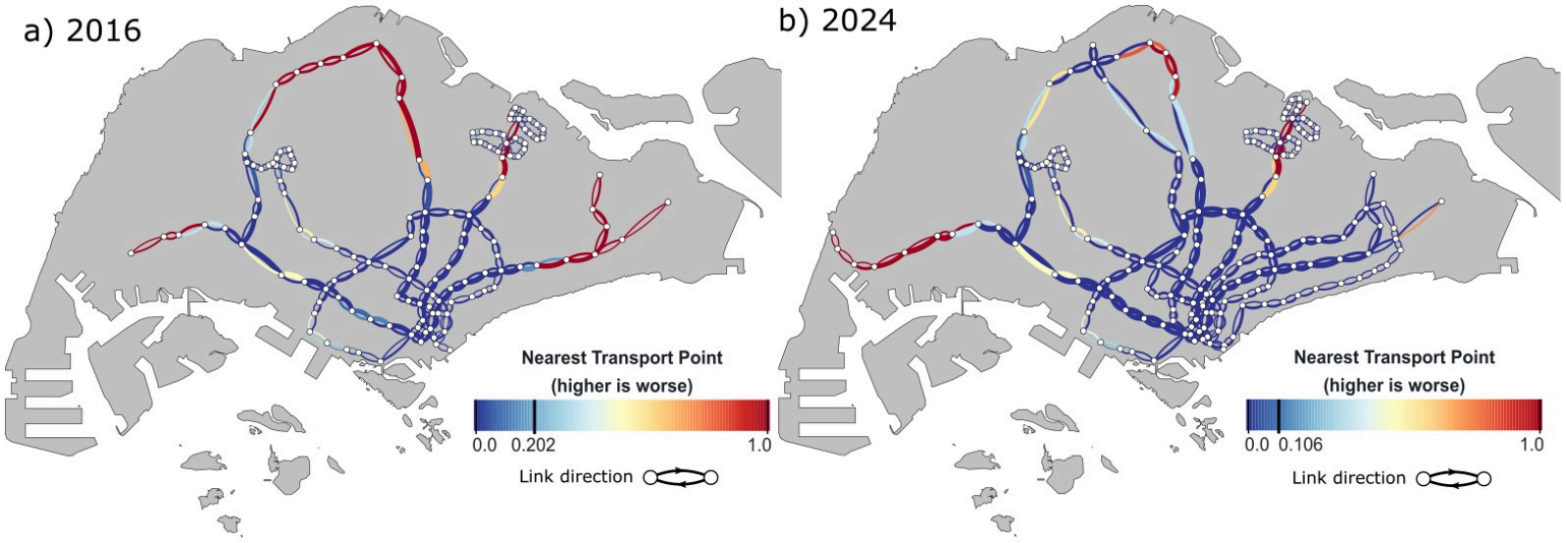
Results of the nearest transport point (NTP) indicator. a) 2016 and b) 2024. For a), the northern and eastern regions of Singapore have low accessibility to alternative lines, giving rise to high NTP scores. These regions are improved by the addition of the TEL and DTL in b), improving NTP scores significantly.

The plots for the NTP indicator shown in Fig 3 indicate a significant stretch of edges having high NTP values in the northern and eastern regions of the 2016 network. This highlights the fact that these regions are less accessible as there are no alternative stations in close proximity in the case of a service disruption. Accessibility in the northern and eastern regions are improved by the presence of the TEL and DTL in 2024 respectively, bringing down the overall flow-weighted average.

**Fig 4:**
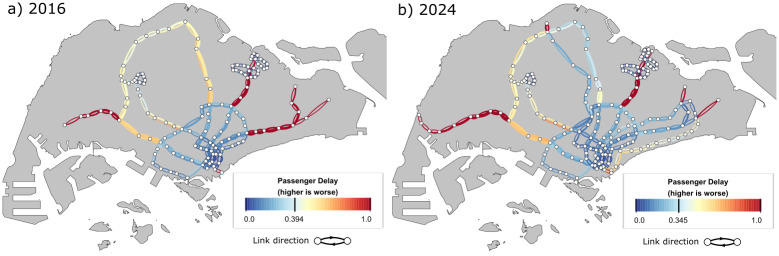
Results of the passenger delay (PD) indicator. a) 2016 and b) 2024. Leaf nodes in the west, east, and northeast regions of a) do not have alternative routes, thus edges in those regions have the maximum PD score of 1. In b), PD scores have improved in the east due to the presence of the TEL and DTL.

The PD scores for the outer portion of the radial lines EWL and NEL are consistently high for the 2016 network as there are no alternative routes when a disruption occurs along those edges, thus all affected passengers passing through those edges are forced to incur the maximum time penalty (refer to Fig 4). For the northern region of Singapore served by the NSL, even though alternative paths exist should an edge be disrupted, commuters have to take a long detour in the opposite direction in order to get to their destination, resulting in moderate to high PD scores. With the TEL providing alternative routes to the city centre in 2024, PD scores with edges in the NSL have improved. Most notably, the TEL and DTL in 2024 provides alternative paths to commuters in the east, reducing delays in journey times for commuters who reroute during disruptions in that region. However, the northeastern and western parts of Singapore remain vulnerable as commuters there still do not have access to alternative routes in the event of disruptions.

**Fig 5:**
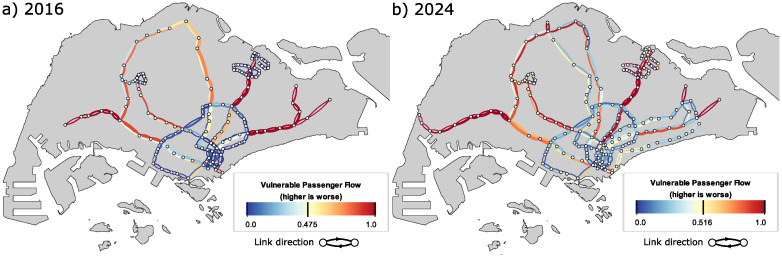
Results of the vulnerable passenger flow (VPF) indicator. a) 2016 and b) 2024. Similar to PD scores, edges without alternative routes are considered vulnerable in VPF computations. In addition, edges that have high passenger flow generally have higher VPF scores as there is a higher probability that these passengers exceed the spare capacity on alternative routes.

Fig 5 plots the scores for the VPF indicator, and it shows some similar spatial characteristics to that displayed by the PD plots—leaf nodes that do not have alternative paths are highlighted as vulnerable areas in both PD and VPF plots. In addition, we observe that the VPF shows high numbers along certain stretches in the 2016 network, even though alternative routes exist. This indicates that those alternative routes are already heavily utilised and there is not much spare capacity available to accommodate redirected commuters should a disruption occur. This is especially evident in the northern region serviced by the NSL as well as edges that are directed towards the city centre.

The addition of the DTL and TEL by 2024 generally reduces the strain on the NSL and the eastern portion of the EWL, by providing alternative routes as well as additional capacity. It is interesting to note the asymmetric behaviour observed in the NSL in 2024—VPF values for the edges pointing in the direction away from the city centre do not seem to benefit directly from the introduction of the TEL compared to the edges that lead to the city centre. One reason is that the TEL runs from the northern region to the eastern region, going through the city centre along the way. Commuters in the north who are heading in the southwest direction would be redirected to an already congested route that passes through the city centre and thus have a high chance of not being able to board.

**Fig 6:**
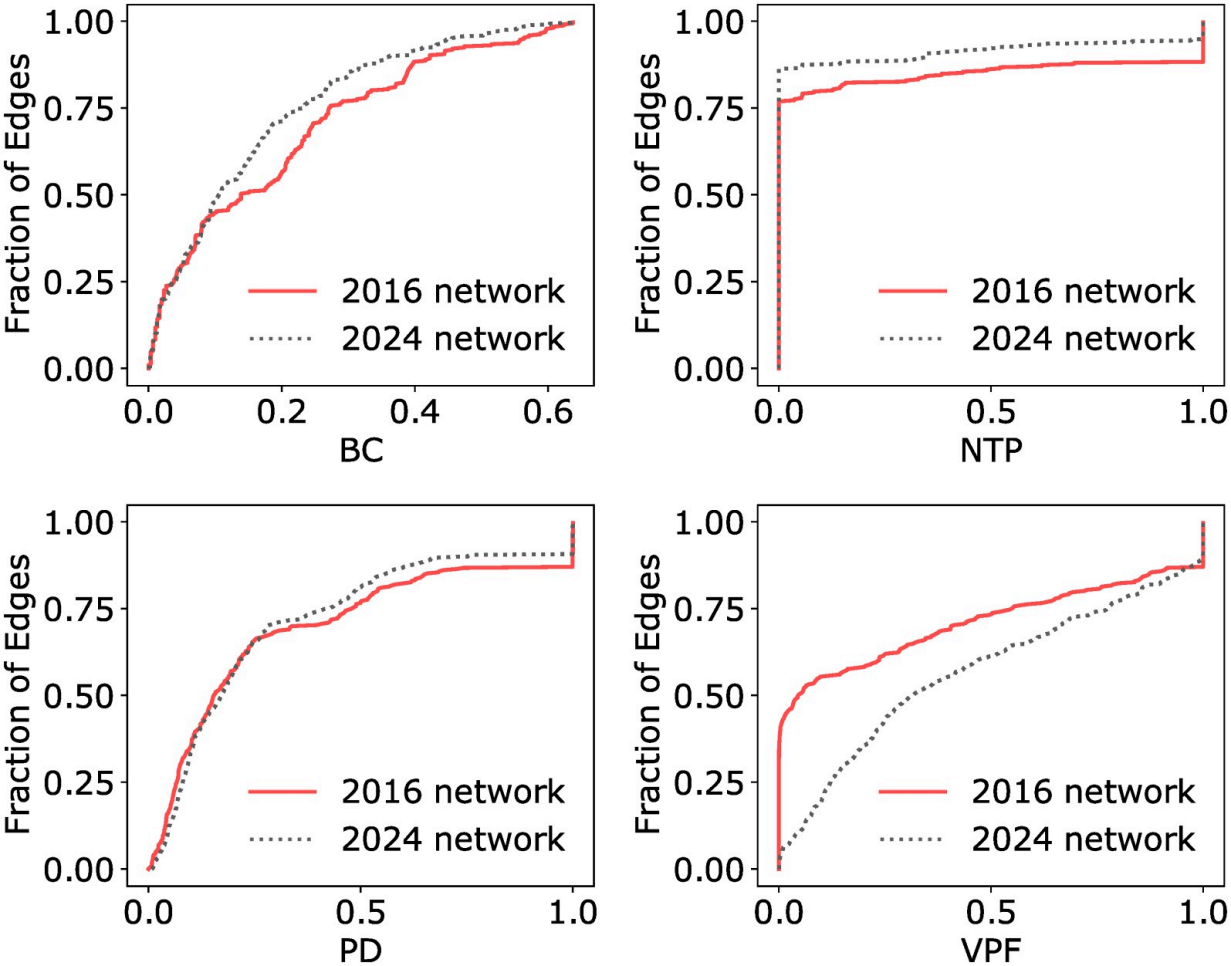
Cumulative distribution function (CDF) plots of the four indicators. The CDF curve for the indicators computed on the 2016 and 2024 network are represented by the solid red line and dotted black line respectively.

Fig 6 visualises the overall changes in the network from 2016 to 2024 by plotting the cumulative distribution function (CDF) curve for each of the four indicators. For a given value of an indicator score (horizontal axis), the CDF curve shows the proportion of edges (vertical axis) that have indicator scores less than or equal to that value. The 2016 and 2024 networks are plotted as solid red and dotted black lines respectively. We observe that for the BC, NTP and PD indicators, the CDF curve for the 2024 network generally lies above that of the 2016 network, indicating that a larger fraction of edges in the 2024 network have a lower (better) indicator score compared to the 2016 network, which is also reflected in the lower flow-weighted averages of the respective indicators.

From the CDF plot and the higher flow-weighted average, VPF is the only indicator that shows an overall increase in value moving from the 2016 to the 2024 network. Assuming that there are no changes in the train frequencies or carrying capacities, this suggests that the estimated growth in commuter demand in 2024 would outpace the planned rail expansion projects and the system would be placed under increased strain by 2024.

Lastly, we computed the Pearson’s product-moment correlation between the four indicators for both the 2016 and 2024 networks and the pair-wise correlation coefficients are shown in Table 2. With the exception of the PD and VPF indicator in 2016, the indicators generally are not strongly correlated with one another. This is because edges without alternative routes are given the same maximum score penalty for both the PD and VPF indicators, and there are more of such cases present in the 2016 network compared to the 2024 network. The low to moderate correlation coefficients indicate that these four indicators are independent measures of different resilience aspects of the network. Additionally, in S2 Appendix and S2 Fig in S1 File, we have also included the pairwise correlation plots of the obtained values of the indicators, further demonstrating their relative independence from each other.

**Table 2:** Pearson product-moment correlation coefficient matrices of the resilience indicators for the 2016 network and 2024 network respectively.

2016 Network					2024 Network				
	BC	NTP	PD	VPF		BC	NTP	PD	VPF
BC	1	0.065	0.33	0.30	BC	1	0.072	0.24	0.087
NTP	—	1	0.64	0.61	NTP	—	1	0.57	0.39
PD	—	-	1	0.90	PD	—	-	1	0.61
VPF	—	-	-	1	VPF	—	-	-	1

### Effect of population growth

**Fig 7:**
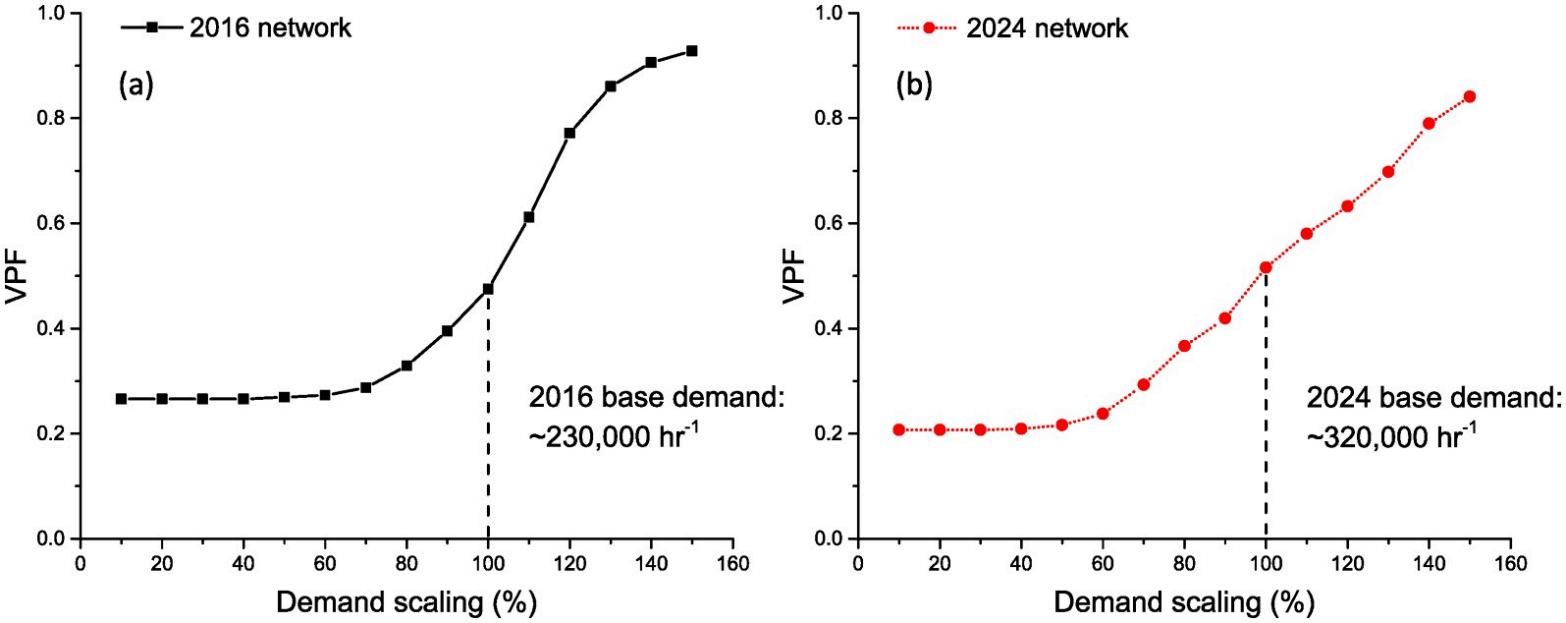
Change in VPF with proportional scaling of commuter population. a) 2016 and b) 2024. Although the VPF indicator is higher in 2024 compared to 2016, the 2016 network shows a much steeper increase in VPF given the same percentage increase in commuter demand past the 100% mark. Lines are drawn between individual simulated values of VPF for different values of *α* as a guides to the eye.

In order to simulate the effect of an increase or decrease in commuter population to the VPF score, we applied a scaling factor *α* to our demand matrix **M**′ = *αM*, while keeping all other parameters constant. Since the population is scaled uniformly across all OD pairs, the three other indicators (BC, NTP, PD) remain constant when population changes. The change in the VPF indicator is shown in Fig 7 for both the 2016 and 2024 network. We observe that the plots for VPF display two regimes separated by a critical value of *α*. Below the critical point, the VPF value remains relatively unchanged with the amount of commuting demand. It can be inferred that the constant VPF value is independent of the amount of spare capacity available and is only dependent on the number of edges in the network which do not have alternative paths. When commuter demand is below this critical value, there is enough spare capacity in alternative routes to accommodate disrupted and redirected commuters and thus VPF is unchanged.

However, above the critical value, the system finds it increasingly difficult to accommodate disrupted commuters as the average loading of the whole system increases, thus reflecting the sharp increase in the VPF indicator value. In addition, we observe that the existing commuter demand (*α* = 100%) for both networks are within the second regime, suggesting that both networks are already strained and would be significantly impacted by disruptions. Conversely, this would also imply that improvements to the train carrying capacities (either by increasing the frequency of trains or modifying the physical trains themselves) would greatly benefit the resilience of the network given this commuter demand.

Lastly, although the VPF indicator shows a slightly higher value for 2024 compared to the 2016 network with their respective commuter demands, the rate at which VPF rises with increasing demand is greater for the 2016 network compared to the 2024 network. This shows that the 2024 network is more robust to increases in population demand in terms of redundant spare capacity.

### Effect of commuter flow redistribution

In this subsection, we investigate how the resilience of a metro network responds to perturbations in the distribution of commuter flow. In general, changes to the pattern of commuter flow can arise when ‘commuter attractors’ such as new job opportunities or tourist attractions get developed, causing commuters to gravitate to these new regions of interest. In the context of Singapore’s metro network during the morning peak period, there is a high flow of passengers towards the central business district (CBD)—the region demarcated by the red circle in Fig 1. In an effort to decentralise and reduce the amount of traffic flowing into the CBD region, urban planners have considered establishing and redirecting commuters to regional centres (RCs) in the eastern and western parts of Singapore (represented by the blue circles in Fig 1).

We study how this would impact the resilience of the metro network by artificially redistributing the commuters towards these RCs, while preserving the total number of commuters. For each OD pair whose destination station falls within the red circle, a percentage *r*% of the commuters gets redirected to a new destination station within the blue circle. OD pairs that experienced shorter average journey time were redirected first. This aims to simulate the effect that commuters who benefited from the policy changes would adapt first. Four separate scenarios were simulated assuming either an eastern or a western RC being developed for either 2016 or 2024. The results for the various indicators are then compared independently. In the subsequent plots, the black squares (red circles) represent redistribution to the eastern (western) RC, while the solid (dotted) lines represent results from the 2016 (2024) network and the different redistribution scenarios are referred to as East(2016), West(2016); East(2024) and West(2024).

**Fig 8:**
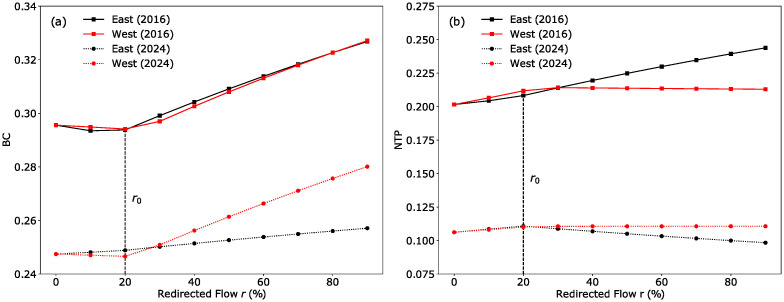
Commuter flow redistribution on betweenness centrality (BC) and nearest transport point (NTP) indicators. (a) There is no significant difference in BC values for the two redistribution scenarios in 2016, while BC rises faster when redirecting commuters to the west compared to the east in 2024. (b) For NTP scores, it is more beneficial when *r* > *r*_0_ to redirect commuters to the west in 2016; in 2024, this trend is reversed and directing commuters to the east results in lower NTP scores.

**Fig 9:**
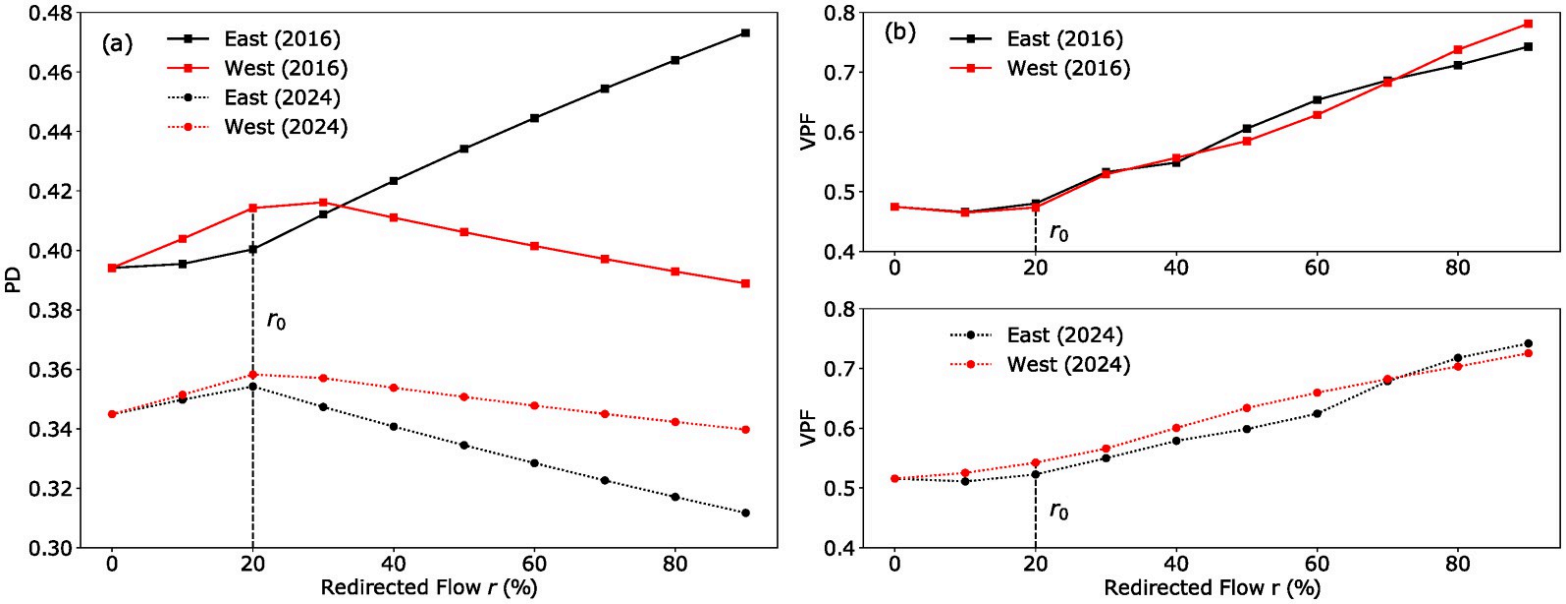
Commuter flow redistribution on passenger delay (PD) and vulnerable passenger flow (VPF) indicators. (a) The PD scores get progressively higher for the 2016 network when more commuters are redirected to the eastern RC due to the lack of alternative paths in that region, in contrast to the 2024 network with the addition of the TEL and DTL. (b) VPF increases with increasing *r* for both networks and scenarios. Similar VPF values obtained for the 2016 network suggest no significant difference between the two redistribution scenarios. For *r* < 70% in the 2024 network, VPF values indicate a slight advantage in directing passengers to the east compared to the west.

Figs 8 and 9 plot the change in the overall BC, NTP and PD value as *r* increases. Several observations can be summarised from these three plots:

- For the 2016 network, redirecting commuters to the east generally results in higher indicator values as compared to redirecting commuters to the west.
- This trend is reversed in the 2024 network, where the eastern RC generally has lower indicator scores than the western RC as *r* increases.
- A critical point is observed in all plots at *r*_0_ ∼ 20%, corresponding to the point where commuters that experience a reduction in travel time due to relocation have all been redirected.

We believe that an overall reduction in BC with commuter redirection can be explained by the fact that the current network configuration is designed to ensure that the CBD region is accessible and robust being well-served by many different lines. Hence, redirecting commuters to RCs with less rail infrastructure generally results in a drop in overall network resilience. This is particularly evident for the East(2016) scenario, where scores across all three indicators increased in value as there is only one line (EWL) serving the eastern RC. Comparing East(2016) against West(2016), the BC indicator shows almost identical behaviour for the two scenarios. On the other hand, NTP and PD scores for West(2016) show a different behaviour—increasing with *r* up to *r*_0_, before decreasing past that point. Results from these three indicators show that the 2016 network is more resilient when redirecting commuters to the western RC over the eastern RC.

In the 2024 network, since there are no new lines in the western region, thus the slope of the indicators for West(2024) are very similar to West(2016). However, since the new lines are directly connected to the eastern RC, the trends observed for the 2016 network are reversed and now the network is more resilient when redirecting commuters to the east compared to the west, with the NTP and PD showing improvement for East(2024).

For both 2016 and 2024 networks, the VPF scores shown in Fig 9 increase as *r* is increased. This appears to be a paradoxical result as we originally expected the network to perform better when commuter loads are more well distributed. However, we concluded that since all of the MRT lines in 2016 and 2024 pass through the CBD region, and these lines provide alternative routes as well as redundant capacity to commuters travelling to the city centre, resulting in low VPF scores around the CBD region (see Fig 5). Since there are fewer lines serving the RCs, redirecting commuters to the RCs would put a heavy strain on those lines, resulting in higher VPF scores as *r* is increased.

For the 2016 network, similar values in the VPF score suggest that there is little difference between directing passengers to the east or west. However, due to opening of new lines in the east for the 2024 network, there is a slight advantage in directing passengers to the east compared to the west when *r* is below 70%.

**Fig 10:**
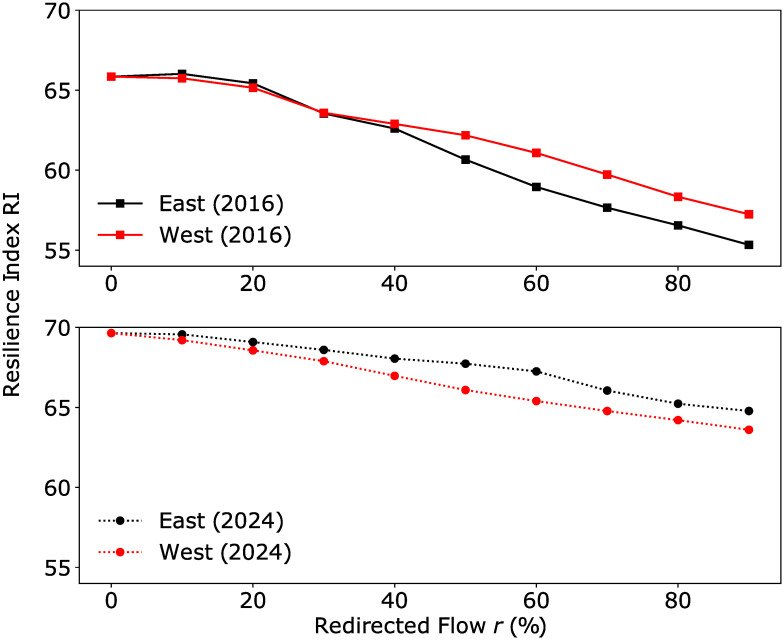
Overall resilience score for commuter redistribution scenarios. Both networks show a decline in resilience as commuters are directed to the RCs, with the 2016 network favouring redirection to the west over the east (smaller drop in resilience score) and vice versa for the 2024 network.

Taking a simple average of the four indicators and converting that number to a percentage according to *RI* = (1 − ∑*wI*/4) × 100%, we obtain an overall score that ranges from 0 (low resilience) to 100 (high resilience) for the network. This is shown in Fig 10 for the different redistribution scenarios. Overall, results from this study indicate that resilience drops as commuters are redirected to RCs in the east or west—with the 2016 network experiencing a larger drop in resilience when commuters are directed to the eastern RC compared to the western RC, and vice versa for the 2024 network.

## Conclusion and future work

In this work, we have developed a framework for quantifying the resilience of RTS networks using a combination of four indicators that encompass various attributes of resilience. This is in contrast to previous studies that usually focus on one aspect of resilience, such as the topological structure of the metro network, which may not give a well-rounded representation of metro resilience. In this framework, a particular indicator evaluates how effectively the metro network can still function if a given edge becomes disrupted. The Betweenness Centrality indicator is a topological-based measure that evaluates the impact of an edge disruption based on the number of shortest paths affected. The Nearest Transport Point indicator uses geographical distance to evaluate the accessibility of switching to another metro line if an edge is disrupted. The Passenger Delay and Vulnerable Passenger Flow indicators evaluate the availability of alternative routes within the metro network in terms of extra commuting time incurred and spare capacity of the alternative routes respectively. These four indicators are designed such that they correspond to physical network flow metrics, while also being normalised so that indicator scores can be aggregated up into an overall score for the entire network. By formulating resilience in such a way, we aim to give a more holistic assessment of metro resilience that can assist policy-makers in their rail planning.

We demonstrated the feasibility of our approach by applying our resilience index formulation on Singapore’s RTS, using historical ticketing records to generate past network configurations which are compared against future network configurations provided by our collaborators. The historical or projected OD demand is first transformed into commuter flows within the network using a multinomial logit flow assignment model. For the BC and NTP indicators, network analysis tools were employed to compute edge betweenness centrality and edge distances. For the PD and VPF indicators, a top-*k* shortest path algorithm was used to generate alternative routes in the event of disruption along an edge. The extra travelling time incurred as well as the spare train capacities along these alternative routes are tracked in order for PD and VPF to be computed. Each indicator is weighted by the commuter flow and the overall resilience score for RTS network in the case study is a simple average of the four indicators. We demonstrated the flexibility of our framework for use in scenario studies such as effects of population growth, or shifts in commuter flow distributions.

There are several limitations to this framework that can be explored in future work. First, this is a macroscopic model where aggregate commuter flows and behaviour are studied. Second, we only consider single-edge disruptions, which is a generalised representation of real-world situations where more than one edge may be disrupted at the same time. Despite these simplifications, we have shown that our formulation can adequately capture and quantify resilience attributes of a RTS network, providing transport planners with another dimension for implementing policies or determining optimal rail alignments.

Finally, as a possible extension of the study, the resilience of a RTS network can be augmented by alternative transportation modes provided by buses or taxis. For example, an indicator that measures the inter-connectivity of RTS stations via existing bus services can be included in the overall resilience computation.

## Supporting information

S1 File (ZIP)

## References

[1] Hughes JF, Healy K. Measuring the resilience of transport infrastructure. NZ Transport Agency; 2014. 546.

[2] Bollinger CR, Ihlanfeldt KR. The impact of rapid rail transit on economic development: the case of Atlanta’s MARTA. Journal of Urban Economics. 1997;42(2):179–204. doi: 10.1006/juec.1996.2020

[3] Legara EF, Monterola C, Lee KK, Hung GG. Critical capacity, travel time delays and travel time distribution of rapid mass transit systems. Physica A: Statistical Mechanics and its applications. 2014;406:100–106. doi: 10.1016/j.physa.2014.02.058

[4] Locatelli G, Invernizzi DC, Brookes NJ. Project characteristics and performance in Europe: An empiricalanalysis for large transport infrastructure projects. Transportation Research Part A: Policy and Practice. 2017;98:108–122.

[5] Pimm SL. The complexity and stability of ecosystems. Nature. 1984;307(5949):321–326. doi: 10.1038/307321a0

[6] Holling CS. Resilience and stability of ecological systems. Annual review of ecology and systematics. 1973;4(1):1–23. doi: 10.1146/annurev.es.04.110173.000245

[7] Rose AZ. Economic resilience to disasters; 2009. http://text2fa.ir/wp-content/uploads/Text2fa.ir-Economic-Resilience-to-Disasters-1.pdf.

[8] Bruneau M, Chang SE, Eguchi RT, Lee GC, O’Rourke TD, Reinhorn AM, et al. A framework to quantitatively assess and enhance the seismic resilience of communities. Earthquake spectra. 2003;19(4):733–752. doi: 10.1193/1.1623497

[9] Bell MG, Iida Y. Transportation network analysis. John Wiley & Sons, Ltd.; 1997.

[10] Berdica K. An introduction to road vulnerability: what has been done, is done and should be done. Transport policy. 2002;9(2):117–127. doi: 10.1016/S0967-070X(02)00011-2

[11] Seeliger L, Turok I. Towards sustainable cities: extending resilience with insights from vulnerability and transition theory. Sustainability. 2013;5(5):2108–2128. doi: 10.3390/su5052108

[12] Carlson J, Haffenden R, Bassett G, Buehring W, Collins III M, Folga S, et al. Resilience: Theory and Application. Argonne National Lab.(ANL), Argonne, IL (United States); 2012.

[13] Musso A, Vuchic VR. Characteristics of metro networks and methodology for their evaluation. National Research Council, Transportation Research Board; 1988.

[14] Gattuso D, Miriello E. Compared analysis of metro networks supported by graph theory. Networks and Spatial Economics. 2005;5(4):395–414. doi: 10.1007/s11067-005-6210-5

[15] Garrison WL, Marble DF. The structure of transportation networks. Northwestern Univ Evanston Ill; 1962.

[16] Kansky KJ. Structure of transportation networks: relationships between network geometry and regional characteristics. University of Chicago; 1963.

[17] Derrible S, Kennedy C. Characterizing metro networks: state, form, and structure. Transportation. 2010;37(2):275–297. doi: 10.1007/s11116-009-9227-7

[18] Derrible S, Kennedy C. The complexity and robustness of metro networks. Physica A: Statistical Mechanics and its Applications. 2010;389(17):3678–3691. doi: 10.1016/j.physa.2010.04.008

[19] Zhang X, Miller-Hooks E, Denny K. Assessing the role of network topology in transportation network resilience. Journal of Transport Geography. 2015;46:35–45. doi: 10.1016/j.jtrangeo.2015.05.006

[20] Wang X, Koç Y, Derrible S, Ahmad SN, Pino WJ, Kooij RE. Multi-criteria robustness analysis of metro networks. Physica A: Statistical Mechanics and its Applications. 2017;474:19–31. doi: 10.1016/j.physa.2017.01.072

[21] Zhang J, Wang S, Wang X. Comparison analysis on vulnerability of metro networks based on complex network. Physica A: Statistical Mechanics and its Applications. 2017;. doi: 10.1016/j.physa.2017.11.137 32288104 PMC7125861

[22] Mattsson LG, Jenelius E. Vulnerability and resilience of transport systems–a discussion of recent research. Transportation Research Part A: Policy and Practice. 2015;81:16–34.

[23] Ramli MA, Monterola CP, Khoon GLK, Guang THG. A Method to Ascertain Rapid Transit Systems’ throughput Distribution Using Network Analysis. Procedia Computer Science. 2014;29:1621–1630. doi: 10.1016/j.procs.2014.05.147

[24] Bell MG. A game theory approach to measuring the performance reliability of transport networks. Transportation Research Part B: Methodological. 2000;34(6):533–545. doi: 10.1016/S0191-2615(99)00042-9

[25] Bell MG, Kanturska U, Schmöcker JD, Fonzone A. Attacker–defender models and road network vulnerability. Philosophical Transactions of the Royal Society A: Mathematical, Physical and Engineering Sciences. 2008;366(1872):1893–1906. doi: 10.1098/rsta.2008.0019 18325875

[26] De-Los-Santos A, Laporte G, Mesa JA, Perea F. Evaluating passenger robustness in a rail transit network. Transportation Research Part C: Emerging Technologies. 2012;20(1):34–46. doi: 10.1016/j.trc.2010.09.002

[27] Rodríguez-Núñez E, García-Palomares JC. Measuring the vulnerability of public transport networks. Journal of Transport Geography. 2014;35:50–63. doi: 10.1016/j.jtrangeo.2014.01.008

[28] Jin JG, Tang LC, Sun L, Lee DH. Enhancing metro network resilience via localized integration with bus services. Transportation Research Part E: Logistics and Transportation Review. 2014;63:17–30. doi: 10.1016/j.tre.2014.01.002

[29] Jenelius E, Cats O. The value of new public transport links for network robustness and redundancy. Transportmetrica A: Transport Science. 2015;11(9):819–835. doi: 10.1080/23249935.2015.1087232

[30] Nian G, Chen F, Li Z, Zhu Y, Sun DJ. Evaluating the alignment of new metro line considering network vulnerability with passenger ridership. Transportmetrica A: Transport Science. 2019;15(2):1402–1418. doi: 10.1080/23249935.2019.1599080

[31] Cats O, Jenelius E. Planning for the unexpected: The value of reserve capacity for public transport network robustness. Transportation Research Part A: Policy and Practice. 2015;81:47–61.

[32] Cats O, Jenelius E. Beyond a complete failure: the impact of partial capacity degradation on public transport network vulnerability. Transportmetrica B: Transport Dynamics. 2018;6(2):77–96.

[33] Cats O, Koppenol GJ, Warnier M. Robustness assessment of link capacity reduction for complex networks: Application for public transport systems. Reliability Engineering & System Safety. 2017;167:544–553. doi: 10.1016/j.ress.2017.07.009

[34] D’Lima M, Medda F. A new measure of resilience: An application to the London Underground. Transportation Research Part A: Policy and Practice. 2015;81:35–46.

[35] Chopra SS, Dillon T, Bilec MM, Khanna V. A network-based framework for assessing infrastructure resilience: a case study of the London metro system. Journal of The Royal Society Interface. 2016;13(118):20160113. doi: 10.1098/rsif.2016.0113 27146689 PMC4892264

[36] Sun D, Guan S. Measuring vulnerability of urban metro network from line operation perspective. Transportation Research Part A: Policy and Practice. 2016;94:348–359.

[37] Sun L, Huang Y, Chen Y, Yao L. Vulnerability assessment of urban rail transit based on multi-static weighted method in Beijing, China. Transportation Research Part A: Policy and Practice. 2018;108:12–24.

[38] Pelletier MP, Trépanier M, Morency C. Smart card data use in public transit: A literature review. Transportation Research Part C: Emerging Technologies. 2011;19(4):557–568. doi: 10.1016/j.trc.2010.12.003

[39] Erlander S, Stewart NF. The gravity model in transportation analysis: theory and extensions. vol. 3. Vsp; 1990.

[40] Simini F, González MC, Maritan A, Barabási AL. A universal model for mobility and migration patterns. Nature. 2012;484(7392):96. doi: 10.1038/nature10856 22367540

[41] Yang Y, Herrera C, Eagle N, González MC. Limits of predictability in commuting flows in the absence of data for calibration. Scientific Reports. 2014;4:5662. doi: 10.1038/srep05662 25012599 PMC4092333

[42] Wang J, Li Y, Liu J, He K, Wang P. Vulnerability analysis and passenger source prediction in urban rail transit networks. PLOS ONE. 2013;8(11):e80178. doi: 10.1371/journal.pone.0080178 24260355 PMC3832441

[43] Cheng YY, Lee RKW, Lim EP, Zhu F. Measuring centralities for transportation networks beyond structures. In: Applications of social media and social network analysis. Springer; 2015. p. 23–39.

[44] McFadden D. Conditional logit analysis of qualitative choice behavior. Frontiers in Econometrics. 1974; p. 105–142.

[45] Monterola C, Legara EF, Pan D, Lee KK, Hung GG. Non-invasive procedure to probe the route choices of commuters in rail transit systems. Procedia Computer Science. 2016;80:2387–2391. doi: 10.1016/j.procs.2016.05.459

[46] Hunt JD. IA LogitModel of Public Transport Route Choice. ITE Journal. 1990;60(12):26–30.

[47] Freeman LC. A set of measures of centrality based on betweenness. Sociometry. 1977; p. 35–41. doi: 10.2307/3033543

[48] Derrible S. Network centrality of metro systems. PLOS ONE. 2012;7(7):e40575. doi: 10.1371/journal.pone.0040575 22792373 PMC3391279

[49] Urban Redevelopment Authority, Accessed February 2022, https://data.gov.sg/dataset/master-plan-2014-region-boundary-no-sea?resource_id=30726aed-9e02-4f3f-88bd-c9f01222cee1

