# Supplementary material for: Quantifying the resilience of rapid transit systems: A composite index using a demand-weighted complex network model
Source: PLoS One. 2022 Apr 28;17(4):e0267222. doi: 10.1371/journal.pone.0267222 (PMC9049543; doi:10.1371/journal.pone.0267222)
Supplement: S1 File — (ZIP) [file pone.0267222.s001.zip › Supporting Information.pdf]

Supporting Information

S1 Appendix. Average BC of a Linear Chain

From Equation 9 of the main text, the BC of an edge e is the proportion of shortest paths that pass through e, divided by the total number of shortest paths for all OD pairs. Since we are studying simple tree graphs with N nodes and $N-1$ edges, there is only one unique shortest path for each OD pair and the equation can be simplified to counting the number of paths that pass through e.

This is illustrated in Fig S1 (a), where an (undirected) tree graph with N nodes is shown. In order to compute the BC for edge e, we note that any route that originates from a node on the left and ends on a node on the right would pass through e, *independent of the network topology* on the left or right of edge e. This implies that the BC depends solely on the number of nodes to the left and right of the edge in question, and for our case, there are altogether $2x\,(N-x)$ number of paths that go through e. Therefore, the BC value for that edge is

Fig S1. Betweenness centrality (BC) of a linear chain. (a) BC of edge e for a simple tree graph only depends on the number of nodes on the left and right of e. (b) Branches in the graph lower the average BC when compared to a linear chain of nodes.

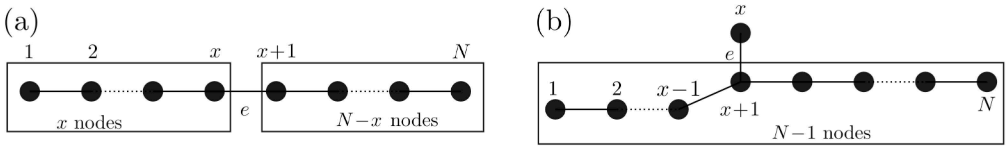

$$BC(e) = BC(x, x+1) = \frac{2x\,(N-x)}{N(N-1)}. \qquad (1)$$

In order to find the maximum BC value, we take the derivative of Equation 1 with respect to x and set it to zero, obtaining the intuitive result of $x = N/2$. In other words, the largest BC of an edge occurs when there are an equal number of nodes on the left and right of that edge. With this result, creating a tree graph with the largest average BC requires a network configuration where the number of nodes on the left and right of each subsequent edge to be as close to each other as possible, and this is only achieved in the linear chain. In Fig S1 (b), the additional branch in the graph reduces the average BC of the network as the edge in that branch only has one node to the 'left' of it when compared to the case if node x is part of a linear chain connecting the $(x-1)^{\text{th}}$ and $(x+1)^{\text{th}}$ node. This can be generalised to an arbitrary number of branches with arbitrary number of nodes on each branch, where each additional branch lowers the average network BC. The network with the lowest average BC is then the network with the maximum number $(N-1)$ of one-node branches, i.e. the star graph.

The average BC for the undirected linear chain BC_0 can be calculated by summing up the BC values for each edge in the network given by Equation 1 and dividing by the total number of edges present:

$$\overline{BC}_0 = \frac{1}{N-1}\sum_{x=1}^{N-1} BC(x, x+1) \\ = \frac{N+1}{3(N-1)}, \tag{2}$$

where we have used summation identities to obtain the final result. For directed graphs, we simply multiply by a factor of 1/2 to obtain the result given in Equation 11 of the main text.

S2 Appendix. Pairwise Scatter Plots for the Resilience Indicators

In Fig S2 , the off-diagonal entries are the pairwise scatter plots of the resilience indicators for each edge of the network, from which the Pearson product-moment correlation coefficients in Table 2 of the main text are computed; while the entries in the main-diagonal are the distributions of indicator values plotted as histograms. From the scatter plots, we observe that the indicators are generally not strongly correlated with one another, with the exception of PD and VPF in the 2016 network. When the structure of the network is modified in 2024, the correlation between PD and VPF is reduced.

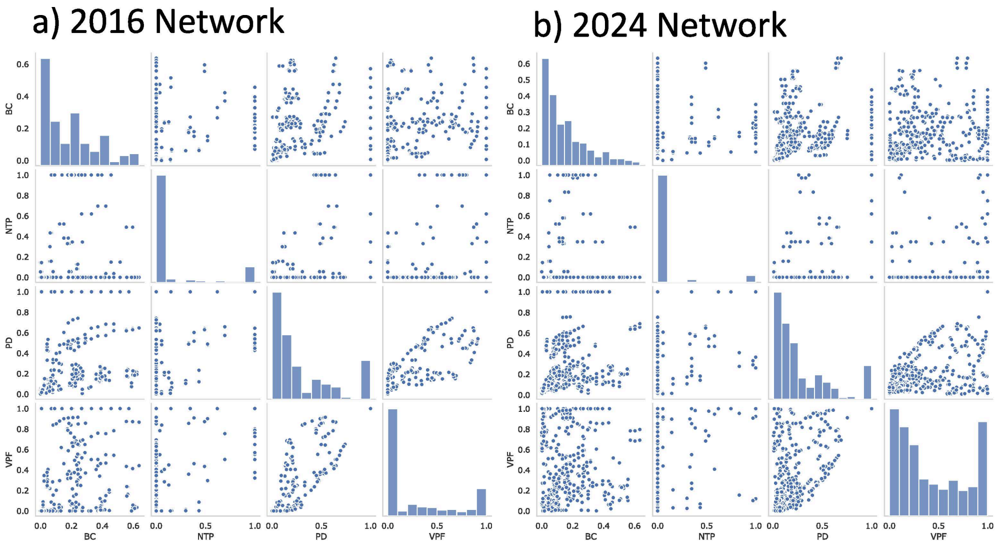

Fig S2. Pairwise scatter plots for the indicators. (a) 2016 network. (b) 2024 network.
